# Somatostatin receptor expression and clinical outcome of multilineage pituitary tumours expressing PIT1 and SF1

**DOI:** 10.1530/EC-23-0328

**Published:** 2023-10-09

**Authors:** Prishila Fookeerah, Winny Varikatt, Meena Shingde, Mark A J Dexter, Mark McLean

**Affiliations:** 1 Department of Diabetes and Endocrinology, Westmead Hospital, Sydney, Australia; 2 School of Medicine, Western Sydney University, Sydney, Australia; 3 Department of Tissue Pathology and Diagnostic Oncology, Westmead Hospital, Sydney, Australia; 4 Westmead Clinical School, University of Sydney, Sydney, Australia; 5 Department of Neurosurgery, Westmead Hospital, Sydney, Australia

**Keywords:** multilineage PIT1 and SF1, pituitary neuroendocrine tumours, pituitary adenomas, acromegaly, somatostatin receptor, transcription factor, plurihormonal

## Abstract

The application of transcription factor immunohistochemistry to pituitary neuroendocrine tumour (PitNET) assessment has allowed identification of tumours that do not conform to a single lineage. Multilineage pituitary transcription factor 1 (PIT1) and steroidogenic factor 1 (SF1) PitNETs are a rare and relatively newly described tumour subtype. These tumours express both transcription factors and may also express combinations of hormones corresponding to both lineages. Histological and clinical characteristics can vary, and overall clinical behaviour and prognosis is not known. We describe the clinical outcomes and somatostatin receptor status (SSTR) of a series of nine cases identified from our cohort of pituitary tumours at Westmead Hospital. Eight PitNETs (88.9%) expressed growth hormone and caused acromegaly at presentation. Of the seven macrotumours that caused acromegaly, one had cavernous sinus invasion. The Ki-67 labeling index score ranged from 0.6% to 3.6%. About 88% of tumours that secreted excess growth hormone exhibited strong immunostaining for SSTR 2 and all tumours displayed weak immunoreactivity for SSTR5. In 62.5% of patients with acromegaly, cure was achieved after surgical resection. Somatostatin receptor ligands resulted in clinical remission in cases where medical treatment was initiated. There was no new tumour recurrence or regrowth over an overall mean follow-up period of 62.5 months.

## Background

The addition of transcription factor immunohistochemistry to pituitary neuroendocrine tumour (PitNET) assessment has resulted in improved detection of tumours arising from different lineages. Most PitNETs demonstrate differentiation to a single-cell lineage, which is characterised by the expression of a single defining transcription factor (steroidogenic factor 1 (SF1) for gonadotrophs, T box transcription factor (TPIT) for corticotrophs and pituitary transcription factor 1 (PIT1) for somatotrophs, lactotrophs and thyrotrophs). The 2022 WHO Classification of Pituitary Tumours recognises two relatively rare exceptions to this rule: (i) multiple synchronous PitNETs; separate tumour populations within a single macroscopic lesion (constituting about 1.2% of all PitNETs) (1) and (ii) plurihormonal PitNETs characterised by the expression of multiple hormones in a monomorphous tumour population (2). However, this second group could also be regarded in terms of demonstration of multiple cell lineages (i.e. expression of more than one transcription factor) rather than expression or secretion of corresponding hormones. Indeed, such tumours might not express any hormones. A recent case series describing plurihormonal PitNETs expressing both PIT1 and SF1 has demonstrated that these tumours can be variable in their histological characteristics and clinical presentation. The authors proposed the new terminology of ‘multilineage PitNETs’ to address the issue of tumours which lack fidelity to a single differentiation pathway (3). Importantly, the long-term clinical outcomes of this relatively new PitNET subtype are not known. In this paper, we describe the histological, clinical and prognostic features of multilineage PIT1 and SF1 tumours identified at our institution.

### Methodology

We retrospectively analysed all available PitNETs resected from 2011 to 2018 and archived at the Institute of Clinical Pathology and Medical Research (ICPMR) at Westmead Hospital. Other sellar masses such as Rathke’s cleft cysts, craniopharyngiomas and pituicytomas were excluded. Recurrent tumours were regarded as one case and therefore only included once. All PitNETs were routinely assessed for all anterior pituitary hormones using immunohistochemistry. For this study, we performed further staining using an automated Ventana platform for transcription factors: TPIT (anti-TPIT antibody CL6251, Abcam), SF1 (anti-SF1 antibody EPR19744, Abcam) and PIT1 (anti-PIT1 antibody ab272639, Abcam). Somatostatin receptor (SSTR) status was examined using anti-SSTR2 (EP149, Epitomics – an Abcam company) and anti-SSTR5 (ab109495, Abcam). Keratins were localised using cytokeratin 8/18 (B221 and B23.1, Cell Marque – a Sigma-Aldrich company). All PIT1-positive tumours were further characterised by applying immunostains for GATA3 and oestrogen receptor (ER). All slides were examined by two investigators to achieve consensus categorisation using the 2022 WHO classification of PitNETs. Intensity of SSTR membranous staining was categorised as weak, moderate or strong, and the extent of tumour staining was defined as diffuse, focal, patchy or variable. Pathology images were captured using an Olympus DP23 camera and acquired using the cellSens Entry software. Clinical data was retrieved using clinicians’ electronic medical records, radiology and pathology reports. Approval was obtained from the Western Sydney Local Health District Human Research and Ethics Committee (approval number: 2021/PID00182).

## Results

After excluding specimens with inadequate tumour tissue for histological assessment, our cohort consisted of 246 PitNETs. Of those, we identified nine PitNETs (3.7%) expressing both PIT1 and SF1. The mean age of patients harbouring these tumours was 44 years (range 24–71 years). There were six male and three female patients. The mean maximum tumour diameter on imaging at diagnosis was 20.9 mm (range 8–38). The mean follow-up duration was 62.5 months (range 12–132 years).

**Figure 1:**
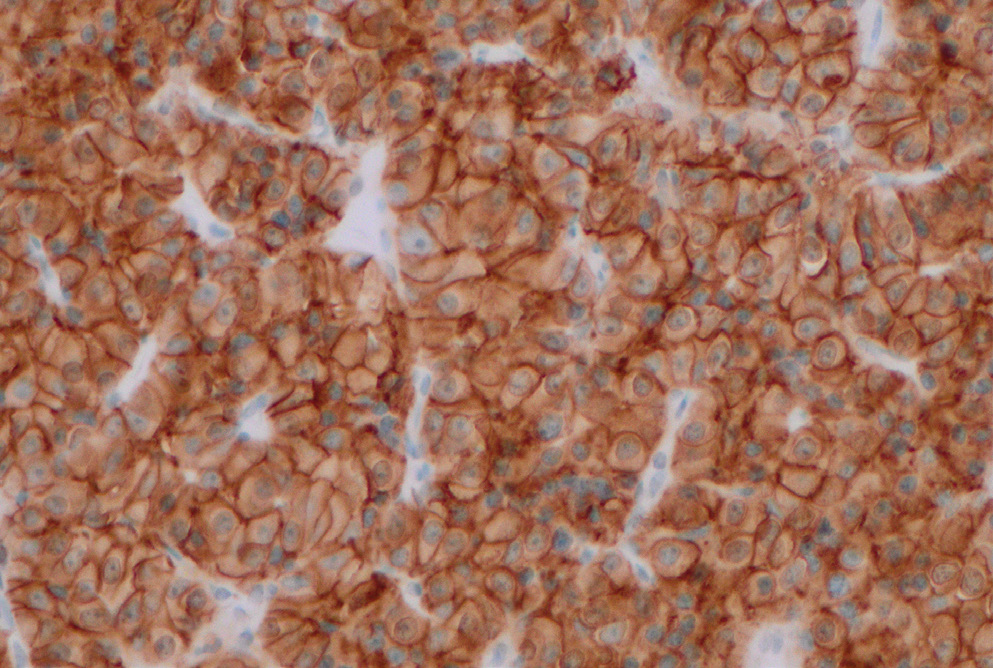
Strong and diffuse SSTR2 immunopositivity (×200 magnification).

**Figure 2:**
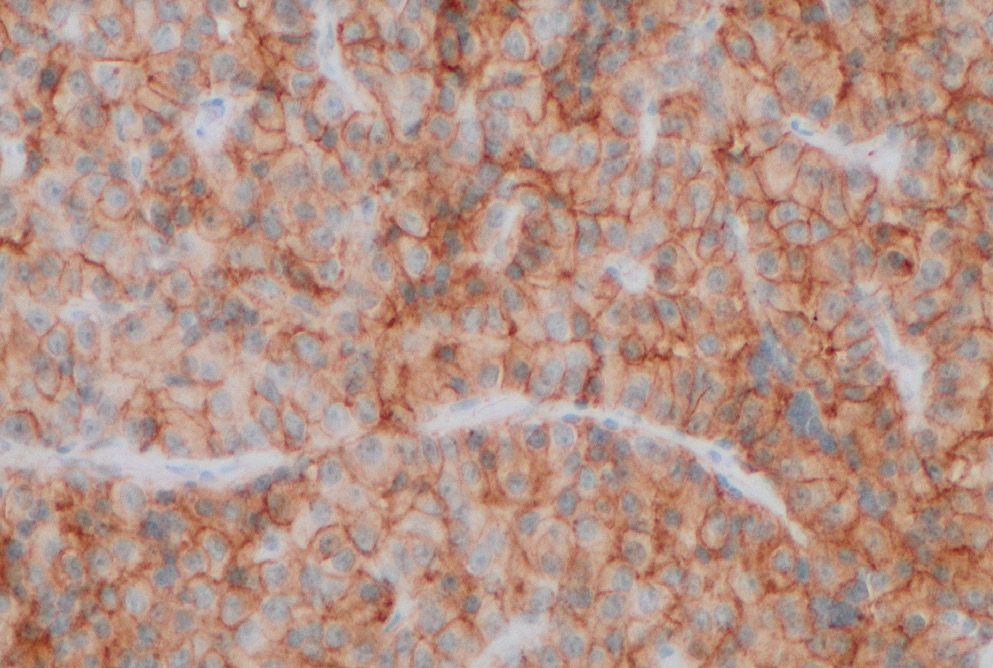
Weak and diffuse SSTR5 positivity seen in most multilineage PIT1 and SF1 tumours causing acromegaly (×200 magnification).

Of the nine tumours, eight presented with acromegaly clinically. One tumour presented with visual field defects and only expressed gonadotrophins on immunostaining although without biochemical or clinical evidence of excess gonadotrophin secretion. All eight tumours causing acromegaly expressed growth hormone (GH) and produced elevated insulin-like growth factor 1 (IGF-1) levels (mean 3.1× upper limit of normal). Of these, six tumours expressed prolactin, but none caused high serum prolactin (PRL) at presentation. One patient had hyperprolactinaemia (4xULN) with amenorrhoea but without PRL immunopositivity. Tumour size was 28 mm at diagnosis, suggesting that the elevated serum prolactin was due to stalk compression resulting in the disinhibition of prolactin secretion from normal pituitary tissue. One tumour co-expressed TSH, GH and PRL without evidence of hyperthyroidism or hyperprolactinaemia clinically or biochemically. The tumours causing GH excess expressed both SSTR2 and SSTR5. SSTR2 immunostaining was strong and diffuse in seven of the eight tumours (Fig. 1), and SSTR5 was present in seven tumours although intensity of staining was weak (Fig. 2). The histological features are summarised in Table 1.

**Table 1:** Histological features of nine PitNETs expressing PIT1 and SF1.

Case	Hormonal expression	SSTR2 expression	Pattern of SSTR2 staining	SSTR5 expression	Pattern of SSTR5 staining	Mitosis (n/10 hpf)	Ki-67 (%)
1	GH, LH	Yes	Strong diffuse	Yes	Weak diffuse	0	1.5
2	GH, LH, FSH	Yes	Strong diffuse	No	–	1	3
3	GH, PRL, TSH, LH	Yes	Weak patchy	Yes	Weak patchy	0	0.6
4	GH, PRL, LH	Yes	Strong diffuse	Yes	Weak diffuse	0	3.3
5	LH, FSH	No	–	No	–	0	3.6
6	GH, PRL, LH	Yes	Strong diffuse	Yes	Moderate diffuse	0	3
7	GH, PRL, LH	Yes	Strong diffuse	Yes	Weak diffuse	0	3.2
8	GH, PRL	Yes	Strong diffuse	Yes	Weak diffuse	0	0.8
9	GH, PRL	Yes	Strong diffuse	Yes	Weak diffuse	0	2.5

In the eight patients with acromegaly, surgical cure was achieved in five (62.5%). In the three cases that did not achieve surgical cure, residual macrotumour (>1 cm) was observed in two, and mild biochemical persistence without visible tumour was observed in one case. The group with surgical cure was followed up for a mean period of 41 months (range 12–59). There was no evidence of radiological or biochemical recurrence. The patient with mild biochemical persistence was treated with a somatostatin receptor ligand (SRL) resulting in normalisation of IGF-1. Of the two residual macrotumours, one was lost to follow-up and the other was managed with SRL with subsequent normalisation of IGF-1.

Only one case did not present with acromegaly and did not express any hormones corresponding to the PIT1 lineage on immunostaining. Clinical presentation was with visual field deficit. PIT1 immunostaining was mostly weak but SF1 staining was strong and diffuse. Staining for GATA3 and ER was positive as seen with gonadotroph PitNETS. This tumour was immunonegative for SSTR2 and SSTR5. A residual macrotumour persisted after surgery without evidence of regrowth over a follow-up period of 34 months. The clinical outcomes for all cases are summarised in Table 2.

**Table 2:** Clinical presentation and outcome of 9 PitNETs expressing PIT1 and SF1.

Case	Presentation	Maximum tumour diameter (mm)	Cavernous sinus or sphenoid sinus invasion	Status after surgery	Follow-up duration (months)	Treatment with SRL	Recurrence or progression
1	Acromegaly	19	No	Cure	48	–	No
2	Acromegaly, amenorrhoea	28	Yes	Residual macrotumour	–	–	–
3	Acromegaly	12	No	Biochemical persistence	129	Yes	No
4	Acromegaly, amenorrhoea	38	No	Residual macrotumour	132	Yes	No
5	Visual field deficit	32	Yes	Residual macrotumour	34	No	No
6	Acromegaly	15	No	Cure	50	–	No
7	Acromegaly	24	No	Cure	59	–	No
8	Acromegaly	12	No	Cure	36	–	No
9	Acromegaly	8	No	Cure	12	–	No

## Discussion

We studied a rare group of PitNETs which has only recently been histologically and clinically characterised. In our cohort, the incidence of PitNETs expressing both PIT1 and SF1 was slightly higher at 3.7%, compared to the previous study reporting 1.3–2.5% (3). Consistent with that recent report, the vast majority of our tumours were presented with acromegaly. All tumours causing acromegaly expressed GH, 75% expressed PRL and one tumour showed immunostaining for TSH. In our cohort, PRL and TSH expression did not seem to correlate with hyperprolactinaemia or central hyperthyroidism clinically. Moreover, these tumours were not always strictly plurihormonal despite expressing transcription factors from two lineages. We therefore concur with the authors’ proposed term of ‘multilineage PitNET’ to define these tumours.

All the eight tumours that caused acromegaly expressed SSTR2 and all but one tumour expressed SSTR5. Previous studies have assessed SSTR expression in acromegaly, but with limited analysis of histological subtypes (4, 5). According to the new 2022 WHO classification of PitNETs, histological subtypes that can cause acromegaly are densely granulated somatotroph tumours, sparsely granulated somatotroph tumours, mature plurihormonal PIT1, immature PIT1 tumours, mammosomatotroph, acidophil stem cell, and mixed somatotroph and lactotroph tumours (2). Here we demonstrate that multilineage PIT1 and SF1 PitNETs can also cause acromegaly, express SSTR and respond to SRL.

**Figure 3:**
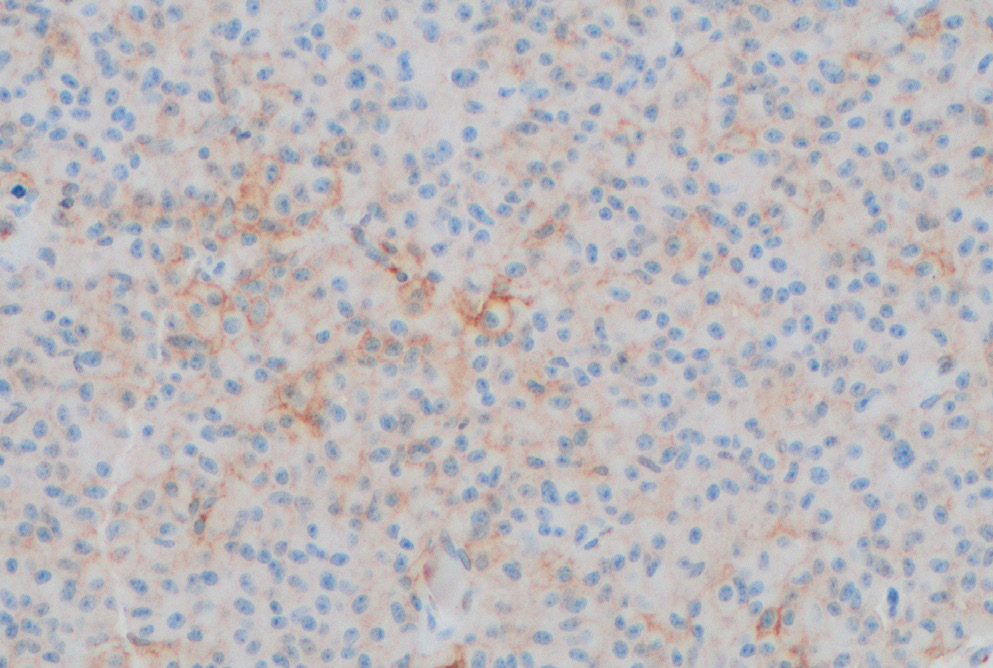
Weak SSTR2 expression in case 3 (×200 magnification).

**Figure 4:**
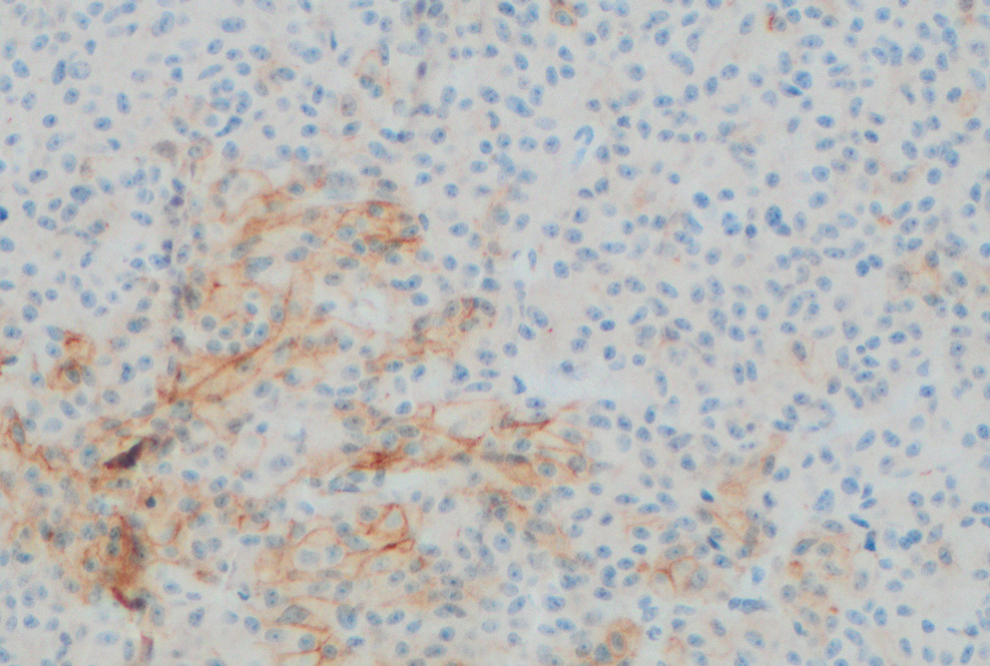
Weak and patchy SSTR5 staining in case 3 (×200 magnification).

The pattern of SSTR expression in this group of tumours appeared to vary. Case 3 had weak membranous immunostaining for SSTR2 (Fig. 3) and patchy staining for SSTR5 (Fig. 4). However, prior treatment with SRL for many months before surgery might have accounted for that appearance (6). All other tumours causing acromegaly exhibited strong diffuse staining for SSTR2, with weaker but positive staining for SSTR5. Consistent with previous literature describing low prevalence of SSTR expression in non-functioning tumours, our non-secreting tumour did not express SSTR 2 or 5 (7, 8).

Importantly, there has not been any indication of recurrence or regrowth in those tumours, indicating relatively good prognosis after surgery and with medical therapy in multilineage PIT1 and SF1 PitNETs. Postoperative cure rate for this group was 62.5%, similar to the findings of other surgical series measuring outcomes in acromegaly (9, 10, 11, 12) but higher than the cure rate for our cohort of pure somatotroph tumours (30%). A Ki-67 index score ≥ 3% was observed in over half of our cases as noted in previous studies (13) but did not correlate with poorer outcome. Two of the nine tumours were radiologically invasive but did not demonstrate clinically aggressive behaviour. Both tumours treated with SRL achieved sustained remission for several years. It is known that tumour SSTR2 expression in acromegaly correlates with lowering of serum IGF-1 and GH levels (14). High SSTR2 expression in this group of tumours causing acromegaly therefore indicates potential therapeutic benefit from SRL if surgery is not an option or does not result in cure.

## Conclusion

We outline the clinicopathological features of a rare PitNET subtype co-expressing PIT1 and SF1. This subgroup of tumours often caused growth hormone excess clinically and highly expressed SSTR in our study. There was no evidence of tumour regrowth or recurrence in any cases of this series. Larger prospective studies are needed to improve our understanding of the nature of these tumours and assist with prognostication in the future.

## Declaration of interest

There is no conflict of interest that could be perceived as prejudicing the impartiality of the research reported

## Funding

This research did not receive any specific grant from any funding agency in the public, commercial or not-for-profit sector.

## Author contribution statement

Study design and supervision by MM and WV. Clinical data provided by MM and MD. Data collection and analysis by WV, PF and MS. Manuscript written by PF with contribution from MM. All authors reviewed and approved the final version of the manuscript.

## References

[1] Mete O Alshaikh OM Cintosun A Ezzat S & Asa SL. Synchronous multiple pituitary neuroendocrine tumors of different cell lineages. Endocrine Pathology 2018 29 332–338. (10.1007/s12022-018-9545-4) 30215160

[2] Asa SL Mete O Perry A & Osamura RY. Overview of the 2022 WHO classification of pituitary tumors. Endocrine Pathology 2022 33 6–26. (10.1007/s12022-022-09703-7) 35291028

[3] Asa SL Mete O Riddle ND & Perry A. Multilineage pituitary neuroendocrine tumors (PitNETs) expressing PIT1 and SF1. Endocrine Pathology 2023 34 273–278. (10.1007/s12022-023-09777-x) 37268858

[4] Rass L Rahvar AH Matschke J Saeger W Renné T Aberle J Flitsch J & Rotermund R. Differences in somatostatin receptor subtype expression in patients with acromegaly: new directions for targeted therapy? Hormones (Athens) 2022 21 79–89. (10.1007/s42000-021-00327-w) 34674191 PMC8818633

[5] Ilie MD Tabarin A Vasiljevic A Bonneville JF Moreau-Grangé L Schillo F Delemer B Barlier A Figarella-Branger D Bisot-Locard S, Predictive factors of somatostatin receptor ligand response in acromegaly-A prospective study. Journal of Clinical Endocrinology and Metabolism 2022 107 2982–2991. (10.1210/clinem/dgac512) 36136828

[6] Casar-Borota O Heck A Schulz S Nesland JM Ramm-Pettersen J Lekva T Alafuzoff I & Bollerslev J. Expression of SSTR2a, but not of SSTRs 1, 3, or 5 in somatotroph adenomas assessed by monoclonal antibodies was reduced by octreotide and correlated with the acute and long-term effects of octreotide. Journal of Clinical Endocrinology and Metabolism 2013 98 E1730–E1739. (10.1210/jc.2013-2145) 24092823

[7] Fuchs TL Sioson L Sheen A Clarkson A & Gill AJ. Immunohistochemical expression of somatostatin receptors SSTR2A and SSTR5 in 299 pituitary adenomas. Pathology 2018 50 472–474. (10.1016/j.pathol.2017.10.024) 29731144

[8] Chinezu L Vasiljevic A Jouanneau E François P Borda A Trouillas J & Raverot G. Expression of somatostatin receptors, SSTR2A and SSTR5, in 108 endocrine pituitary tumors using immunohistochemical detection with new specific monoclonal antibodies. Human Pathology 2014 45 71–77. (10.1016/j.humpath.2013.08.007) 24182563

[9] Gittoes NJ Sheppard MC Johnson AP & Stewart PM. Outcome of surgery for acromegaly--the experience of a dedicated pituitary surgeon. QJM 1999 92 741–745. (10.1093/qjmed/92.12.741) 10581337

[10] Kim MS Jang HD & Kim OL. Surgical results of growth hormone-secreting pituitary adenoma. Journal of Korean Neurosurgical Society 2009 45 271–274. (10.3340/jkns.2009.45.5.271) 19516943 PMC2693785

[11] Nomikos P Buchfelder M & Fahlbusch R. The outcome of surgery in 668 patients with acromegaly using current criteria of biochemical 'cure'. European Journal of Endocrinology 2005 152 379–387. (10.1530/eje.1.01863) 15757854

[12] Shimon I Cohen ZR Ram Z & Hadani M. Transsphenoidal surgery for acromegaly: endocrinological follow-up of 98 patients. Neurosurgery 2001 48 1239–1244. (10.1097/00006123-200106000-00008) 11383725

[13] Mete O Cintosun A Pressman I & Asa SL. Epidemiology and biomarker profile of pituitary adenohypophysial tumors. Modern Pathology 2018 31 900–909. (10.1038/s41379-018-0016-8) 29434339

[14] Gatto F Arvigo M & Ferone D. Somatostatin receptor expression and patients' response to targeted medical treatment in pituitary tumors: evidences and controversies. Journal of Endocrinological Investigation 2020 43 1543–1553. (10.1007/s40618-020-01335-0) 32557353

